# Covid-19 vaccination among migrants in Rome, Italy

**DOI:** 10.1038/s41598-023-48273-4

**Published:** 2023-11-28

**Authors:** Laura Cacciani, Giulia Cesaroni, Enrico Calandrini, Marina Davoli, Nera Agabiti

**Affiliations:** Department of Epidemiology of the Regional Health Service of Lazio, Asl Roma 1, Rome, Italy

**Keywords:** Risk factors, Infectious diseases, Epidemiology, Disease prevention

## Abstract

Migrants may be susceptible to vaccine barriers and hesitancy. We evaluated the association between migrant status, as measured by the citizenship from a High Migratory Pressure Country (HMPC), and COVID-19 vaccination uptake in the resident population in Rome, Italy. We also investigated sex differences. We followed participants for vaccination against COVID-19 in 2021. We calculated crude- and adjusted-vaccination rates and Cox hazard ratios of vaccination for migrants compared to Italians. Among migrants from HMPCs, we estimated HRs for females compared to males, stratifying by geographical area of origin. Models were adjusted for age and deprivation index and stratified by infection history. In 2021, among 1,731,832 18–64-year-olds, migrants were 55% less likely to uptake at least one COVID-19 vaccine dose than their Italian counterpart. Past SARS-CoV-2 infection reduced the difference between migrants and Italians to 27%. Among migrants from HMPCs, we observed a slight excess of vaccination uptake among females compared to males. Focusing on geographical areas, we observed that only females from central-western Asia were 9% less likely to uptake vaccination than males. Health communication strategies oriented to migrants and considering their different languages, cultures, and health literacy should be adopted for prevention before emergencies.

## Introduction

There is evidence that some refugees and migrants in Europe may be susceptible to vaccine barriers and hesitancy^1,2^, and that they have lower immunization rates compared to European-born individuals^3^. In an Italian multi-centre retrospective cohort study, lower paediatric vaccination coverage was observed for children born to mothers from HMPCs compared to Italy plus Advanced Developed Countries in Rome^4^. Studies based on vaccination registers, integrated with other variables on the migrant-status and possibly with socio-demographic information, would help to produce evidence-based results on disparities of vaccination uptake among migrants and refugees, supporting decisions on interventions to increase their access to vaccination uptake, following with the statement published by the Committee on Bioethics of the Council of Europe: “COVID-19 and vaccines: Ensuring equitable access to vaccination during the current and future pandemics”^5^.

Low vaccination uptake among migrants is striking as there is evidence that this population has been primarily affected by the COVID-19 pandemic in many countries. In a review conducted in 2020, migrants in high-income countries have been found to be at high risk of exposure to and infection with COVID-19^6^. Multicentric Italian studies have documented the impact of the pandemic on migrants: compared to Italians, migrants have had higher overall and intensive care unit hospitalization rates^7^, as well as higher incidence and mortality, but only in some phases of the pandemic^8,9^. Other Italian studies found higher hospitalization and mortality among non-Italian nationals with COVID-19^10,11^, a much higher proportion of not vaccinated in foreign-born (25.4%) than in Italian-born (9.0%)^12^, and a three-fold COVID-19 adjusted vaccination coverage among Italians plus foreigners from Advanced Developed Countries compared to HMPCs^13^. In Rome, there is a high percentage of foreign residents (13.3%)^14^, in particular migrants coming from HMPCs, which are the most prevalent and disadvantaged component of the foreign population, deserving attention from a public health perspective. Therefore, to add evidence on COVID-19 vaccination uptake among migrants, in the present retrospective study, we evaluate the association between migrant status, as measured by the citizenship from an HMPC, and COVID-19 vaccination uptake in the resident population in Rome, Italy. We also investigate differences between males and females among HMPCs.

## Results

### Participants

In December 2020, there were 2,823,659 individuals residing in Rome (target population), and 2,818,090 (99.8%) had the anonymous identifier useful for record-linkage with health archives. Among those, 1,731,832 (61.5%) 18–64-year-old individuals were eligible for this study (study population). Therefore, we analysed 1,731,595 subjects (99.9%) with information on citizenship. In our study population, 1,714,552 individuals (99.0%) completed the follow-up, while 17,043 (1.0%) were assumed to have a 6-month follow-up time.

### Descriptive data

Table 1 describes the main characteristics of the study population by migrant status (the primary exposure variable). People from HMPCs represented 16% of our population and were the youngest (mean age 41.6 years); the prevalence of females was higher among HMPCs (53%) and High Developed Countries (HDCs, 61%) compared to Italy (51%). The deprivation index (DI) indicated a higher percentage of disadvantaged adults among HMPCs (23%) compared to Italy (18%) and HDCs (12%). People from HDCs had the highest percentage of SARS-CoV-2 infection (48%).

**Table 1:** Characteristics of the resident population by citizenship. Rome, Italy, December 2020.

	Italy		HDCs		HMPCs		Total	
	n = 1,444,925		n = 17,595		n = 269,075		n = 1,731,595	
	n	Column %	n	Column %	n	Column %	n	Column %
Sex								
Females	738,996	51.1%	10,819	61.5%	141,945	52.8%	891,760	51.5%
Males	705,929	48.9%	6776	38.5%	127,130	47.2%	839,835	48.5%
DI (quintiles)								
Missing	93,605	6.5%	1332	7.6%	25,100	9.3%	120,037	6.9%
1	279,714	19.4%	4826	27.4%	37,585	14.0%	322,125	18.6%
2	279,116	19.3%	3818	21.7%	39,398	14.6%	322,332	18.6%
3	272,775	18.9%	3038	17.3%	46,684	17.3%	322,497	18.6%
4	261,344	18.1%	2384	13.5%	58,339	21.7%	322,067	18.6%
5	258,371	17.9%	2197	12.5%	61,969	23.0%	322,537	18.6%
SARS-CoV-2 infection								
Yes	168,719	11.7%	8506	48.3%	97,876	36.4%	275,101	15.9%
No	1,276,206	88.3%	9089	51.7%	171,199	63.6%	1,456,494	84.1%
Age (yrs.), mean (sd)	43.8	(13.1)	45.1	(11.0)	41.6	(11.3)	43.5	(12.8)

Participants totalized 916,213 years of follow-up. Table 2 summarises follow-up times (days) by citizenship. On average, people were followed for 193 days. Median follow-up times among non-Italians (228 days for HDCs and 222 for HMPCs) were higher than among Italians (164 days).

**Table 2:** Follow-up time of study participants by citizenship.

Citizenship	Participants	Follow-up time (days)					
	N Obs	Sum	Mean	Std Dev	Median	Min	Max
Italy	1,444,925	262,524,407	182	88	164	0	369
HDCs	17,595	4,437,014	252	115	228	3	369
HMPCs	269,075	67,685,451	252	95	222	0	369
Total	1,731,595	334,646,872	193	93	170	0	369

### Outcome data

**Figure 1:**
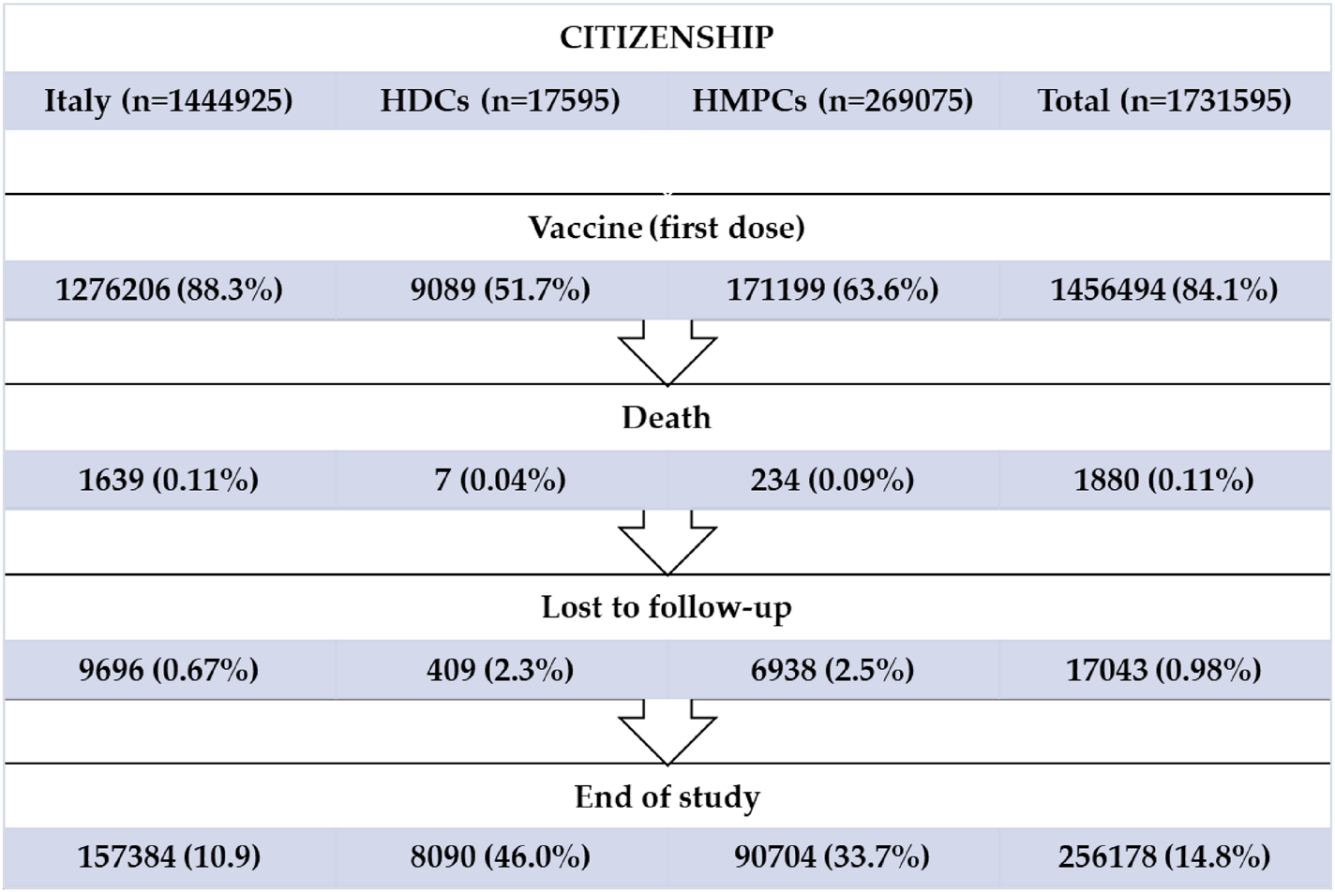
Outcome data by citizenship.

Figure 1 shows the outcome data and reasons for censoring by citizenship. Out of 1,731,595 individuals analysed, Italians had the highest percentages of people vaccinated with at least one vaccine dose (88.3%), while non-Italians coming from HDCs showed the lowest percentages of vaccination uptake (51.7%). Reasons for censoring were due either to death for 1880 individuals (0.11%) or loss to follow-up for 17,043 individuals (0.98%), while 256,178 (14.8%) completed the follow-up until the end of the study.

Time to vaccination distributions were slightly right-skewed and showed a higher median time among people from HMPCs (181 days) compared to Italians (159 days) and people from HDCs (153 days).

### Rates

#### Migrant status

Crude vaccination rates (per person-year) were lower among people of HMPCs (VR = 0.920; 95%IC 0.916–0.924) than Italians (VR = 1.766; 95%IC 1.763–1.769). Adjustments for age and DI did not influence the vaccination uptake much. The history of past infection influenced the rates: age- and DI-adjusted VR were lower among people of HMPCs without previous infections (VR = 0.941; 95%CI 0.937–0.946) compared to those with previous infection (VR = 1.251; 95%CI 1.228–1.274); among Italians, we observed an opposite pattern that showed higher vaccination rate among those without previous infection (VR = 1.808; 95%CI 1.805–1.812) compared to those with previous infection (VR = 1.547; 95%CI 1.538–1.556) (Table 3).

**Table 3:** Crude and adjusted vaccination rates (per person-year) by citizenship and past SARS-CoV-2 infection.

	HMPCs		Italy	
	Rate	95% CI	Rate	95% CI
Crude				
Overall	0.920	0.916, 0.924	1.766	1.763, 1.769
By past infection				
No	0.903	0.898, 0.907	1.796	1.793, 1.800
Yes	1.218	1.197, 1.239	1.536	1.528, 1.544
Age-adjusted				
Overall	0.936	0.931, 0.940	1.540	1.531, 1.548
By past infection				
No	0.919	0.915, 0.924	1.801	1.798, 1.805
Yes	1.225	1.203, 1.246	4.216	4.193, 4.239
Age- and DI-adjusted				
Overall	0.958	0.953, 0.963	1.778	1.775, 1.781
By past infection				
No	0.941	0.937, 0.946	1.808	1.805, 1.812
Yes	1.251	1.228, 1.274	1.547	1.538, 1.556

#### Sex

Among people of HMPCs, crude vaccination rates (per person-year) were higher among females (VR = 0.948; 95%IC 0.942–0.954) than males (VR = 0.890; 95%IC 0.884–0.897). Adjustments for age and DI did not change the vaccination uptake much. However, history of past infection increased the vaccination uptake both among females (age- and DI-adjusted VR = 1.237; 95%CI 1.207–1.268) and males (1.267; 95%CI 1.232–1.303) compared to those without previous infection (0.935; 95%CI 0.929–0.942, females; 0.910; 95%CI 0.903–0.917, males) (Table 4).

**Table 4:** Crude and adjusted vaccination rates (per person-year) for people of HMPCs by sex and past SARS-CoV-2 infection.

	Females		Males	
	Rate	95% CI	Rate	95% CI
Crude				
Overall	0.948	0.942, 0.954	0.890	0.884, 0.897
By past SARS-CoV-2 infection				
No	0.942	0.924, 0.937	0.873	0.866, 0.879
Yes	1.215	1.187, 1.244	1.221	1.189, 1.253
Age-adjusted				
Overall	0.942	0.937, 0.949	0.898	0.892, 0.904
By past SARS-CoV-2 infection				
No	0.925	0.919, 0.931	0.880	0.874, 0.886
Yes	1.212	1.184, 1.241	1.229	1.197, 1.261
Age- and DI-adjusted				
Overall	0.953	0.946, 0.960	0.928	0.921, 0.935
By past SARS-CoV-2 infection				
No	0.935	0.929, 0.942	0.910	0.903, 0.917
Yes	1.237	1.207, 1.268	1.267	1.232, 1.303

### Regression models

#### Migrant status

We performed Cox regression models among people from HMPCs and Italians. The association between migrant status and vaccination (dependent variable) showed a lower vaccination hazard among migrants than Italians (HR = 0.440; 95%CI 0.438–0.442). Age and DI adjustment did not change the observed associations (HR = 0.451; 95%CI 0.448–0.453).

Table 5 shows unadjusted, age-, and age-DI-adjusted HRs stratified by past SARS-CoV-2 infection. Past infection was an effect modifier: among participants without past infection, migrants showed a lower hazard of being vaccinated compared to Italians (HR = 0.432; 95%CI 0.429–0.434) that got closer to the hazard for Italians among participants with past infection (HR = 0.733; 95%CI 0.719–0.747). The interaction term tested in the model was statistically significant (p < 0.0001). We did not find a relevant interaction between either sex or age and migrant status.

**Table 5:** Association between migrant status and vaccination by past SARS-CoV-2 infection: results from Cox regression models.

	Crude model		Age-adjusted model		Age- and DI- adjusted model	
	HR	95% CI	HR	95% CI	HR	95% CI
No past infection						
HMPCs (ref = Italians)	0.421	0.419, 0.424	0.423	0.421, 0.425	0.432	0.429, 0.434
Age (year)			1.023	1.023, 1.023	1.023	1.023, 1.023
DI quintile (ref = 1)						
2					0.974	0.969, 0.980
3					0.925	0.920, 0.930
4					0.887	0.882, 0.892
5					0.787	0.782, 0.791
Past infection						
HMPCs (ref = Italians)	0.716	0.703, 0.729	0.717	0.705, 0.731	0.733	0.719, 0.747
Age (year)			1.013	1.012, 1.013	1.013	1.012, 1.013
DI quintile (ref = 1)						
2					0.986	0.969, 1.003
3					0.929	0.913, 0.945
4					0.877	0.862, 0.892
5					0.791	0.777, 0.804

#### Sex

We performed Cox regression models only among people from HMPCs. Overall, the association between sex and vaccination (dependent variable) showed a higher risk of being vaccinated among women than men (HR = 1.110; 95%CI 1.100–1.121). Age and DI were not relevant confounders of the observed associations (HR = 1.049; 95%CI 1.039–1.060). When stratified by past infection, such differences between females and males disappeared among those with a history of past infection (Table 6). The interaction term tested in the model was statistically significant (p < 0.01).

**Table 6:** Association between sex and vaccination by past SARS-CoV-2 infection among people of HMPCs: results from Cox regression models.

	Crude model		Age-adjusted model		Age- and DI-adjusted model	
	HR	95% CI	HR	95% CI	HR	95% CI
No past infection						
Sex (ref = males)	1.115	1.104, 1.126	1.081	1.071, 1.092	1.052	1.041, 1.063
Age (year)			1.011	1.011, 1.011	1.011	1.010, 1.011
DI quintile (ref = 1)						
2					1.018	1.000, 1.037
3					1.004	0.986, 1.022
4					0.978	0.962, 0.995
5					0.871	0.856, 0.886
Past infection						
Sex (ref = males)	0.997	0.963, 1.033	0.983	0.949, 1.018	0.968	0.933, 1.004
Age (year)			1.013	1.012, 1.015	1.013	1.011, 1.015
DI quintile (ref = 1)						
2					0.972	0.908, 1.040
3					0.914	0.857, 0.974
4					0.828	0.779, 0.880
5					0.780	0.734, 0.829

The focus on each geographical area of origin showed a different pattern among people of central-western Asia (where 86% of people came from Bangladesh, India, Sri Lanka, and Pakistan), with women having a lower age-DI-adjusted hazard of being vaccinated compared to men (HR = 0.911; 95%CI 0.890–0.932). We did not observe differences according to past infections.

#### Additional analysis

When we selected the participants, excluding those who had an infection before 27 December 2020 (n = 1,658,686), and we truncated the follow-up time at the date of infection for those who got infected in 2021 (n = 72,881), we found comparable results with those presented above: the migrant status was inversely associated to the COVID-19 vaccination (HR = 0.436; 95%CI 0.434–0.439) and women had a higher probability than men of getting the vaccine (HR = 1.051; 95%CI 1.040–1.062). The results were comparable for central-western Asian women (HR = 0.911; 95%CI 0.890–0.933).

## Discussion

In 2021, among 1,731,832 residents in Rome aged 18–64, migrants from HMPCs were 55% less likely to uptake at least one COVID-19 vaccine dose than their Italian counterpart, independently of age and area deprivation index. Past SARS-CoV-2 infection reduced the difference between migrants and Italians to 27%, explained by an increase in vaccination uptake after the infection among migrants and a decrease among Italians. Among migrants from HMPCs, we observed a slight excess of vaccination uptake among females compared to males; while, focusing on geographical areas of origin we did observe that only females from central-western Asia were 9% less likely to uptake vaccination than males. The additional analysis showed comparable results.

Some limitations should be considered. Suppose there were different patterns of vaccination uptake, inside or outside the region, between migrants and Italians. In that case, the observed associations might be biased as we accessed only data from Lazio (the region of Rome). Mobility among migrants may be higher than among non-migrants, which would cause an underestimate of vaccination coverage and bias the observed hazard ratios toward the lower bound. Nevertheless, we studied the resident population, and resident migrants are likely less prone to mobility than non-resident migrants. In addition, due to pandemic restrictions, it is likely that during 2021 mobility was reduced. Another limitation may be related to the record-linkage procedures that could be less efficient among the migrant population than Italians. This would again yield an underestimation of vaccination uptake among migrants. Concerning the adjustment for the deprivation index, based on 2011 Census data, any change in the social tissue that occurred over ten years might imply misclassification. In relation to other sources of confounding, we could adjust only for major factors (age, DI), while other factors, like comorbidities, occupation, or marital status, might also play a role. Finally, contextual factors changing over time, such as fluctuating mobility restrictions, risk of infection, or policies oriented to mandatory vaccination, might directly or indirectly affect the associations. However, the interpretation of models that include calendar time, with cut-offs appropriate for each factor, would be complex. As such, we decided not to adjust or stratify for calendar time in the present study.

The results indicate the vulnerability of migrants residing in Rome concerning COVID-19 vaccination access in 2021 and suggest inequalities in health. Lower vaccination uptake has also been observed in an Italian study conducted in Lazio, the administrative region of Rome, which found that foreign residents have a triple probability of Italians not accessing the vaccine^12^, and in a study conducted in a Local Health Authority in Rome^13^. Furthermore, a lower vaccination coverage among foreigners has also been observed in a study conducted in Brescia Province, one of the most dramatically hit by COVID-19 at the beginning of the pandemic^15^. To interpret the findings, we should consider that COVID cases among migrants might have different characteristics affecting the risk of infection and the need to be vaccinated. Among these, later diagnoses and poorer outcomes in COVID cases among foreigners compared to Italians were reported^10,11^. In the study conducted by a Local Health Authority in Rome, HMPC citizens were younger than Italians, less likely to be frail and more likely to receive the less effective brand of vaccine (Janssen)^13^. However, we do not have striking evidence of different risks of infection among immigrants in Italy, though it is possible to argue that the lack of findings is the consequence of the lower access to diagnostic tests^16,17^. Concerning the young age structure of migrants, in our study, we selected 18–64-year-olds, reducing the age gap between Italians and immigrants (mean age 43.8 vs 41.6, Table 1) and, as such, differences in frailty. At the international level, several studies analysed the association between ethnicity and vaccination uptake, using different study designs and measures from our own. For example, in a study conducted over 24 million adults in England, the first dose of COVID-19 vaccination was lower among all ethnic minority groups compared with white British adults^18^. Another study conducted in Denmark over 4.9 million individuals aged 12 years or more in 2021 found that non-vaccination was most pronounced among migrants or descendants^19^. In contrast, a study conducted in Switzerland did not find an association between Swiss-born and foreign-born individuals^20^. We also observed that women from central-western Asia showed lower vaccination coverage than men. In this area, the most prevalent origin countries of subjects living in Rome are Bangladesh, India, Sri Lanka, and Pakistan, and most subjects are males. Most central-western Asian women typically come to Italy for family reunification. They are often unemployed and have few social contacts^21^. All these aspects may partly explain the lower vaccination coverage among women in this subgroup of migrants.

Various factors in the literature have been identified as possible explanations for low vaccine uptake and hesitancy, for example, the “delay in acceptance or refusal of vaccines despite availability of vaccination services”^22^ among migrants. A systematic review exploring barriers and facilitators of vaccine uptake has identified language, literacy, communication, practical, legal, and service barriers in the uptake of vaccines^1^. In our country, access barriers to health services have been identified for migrants^23^. These barriers also played a role in the vaccination uptake, especially at the beginning of the vaccination campaign. Later, on 15 October 2021, the vaccine became mandatory for all people over 50 years and for occupied people. Then, the possession of a green pass, a document certifying the vaccination, was imposed at work to demonstrate full vaccination coverage^24^. According to the 3C model on vaccine hesitancy developed by the SAGE Working Group, three main factors influence vaccine uptake: confidence, complacency, and convenience barriers^22^. In a recent systematic review performed to synthetise qualitative studies on the reasons for vaccine hesitancy among migrants, the Authors found the “confidence” dimension of the 3C model, that is, “…people are vaccine hesitant because they have low confidence in the vaccine’s effectiveness and safety and distrust scientists, policymakers and health professionals…”^22^, represents a disproportionately large barrier to vaccine uptake in ethnic minority groups^25^. We argue that the confidence dimension may explain vaccine hesitancy among the migrants in Italy because communication during the vaccination campaign was challenging due to linguistic barriers and the different health literacy of migrants compared to Italians, despite some communication strategies adopted in the country^26^. Health literacy may be associated with vaccination, although evidence is scarce^27^. In addition, the “convenience” dimension, that is “…people are vaccine hesitant because there are a number of barriers (physical, logistical or economical) that hinder them from getting a vaccine…”^22^, may have represented another important explanation for the vaccine hesitancy among migrants in Italy. In fact, it is already documented that migrants encounter, in Italy^23,28^ as in other European countries^29^, various barriers in accessing health services that during the pandemic may have represented a critical issue for COVID-19 prevention^30,31^. In addition, we argue that since migrants in Italy are often employed in temporary and precarious jobs^32,33^, their intention to be vaccinated may be undermined by the fear of possible vaccine collateral effects limiting their chance to work. The ECDC suggests various approaches to strengthening vaccine uptake in migrants. Some of them may be particularly relevant in Italy and adopted, such as the provision of “…simple, accurate culturally-relevant resources about the COVID-19 vaccine in a range of languages, literacy levels and formats…” and the provision of cultural mediators in primary care^34^. In addition, the availability of data stratified by origin country and other relevant factors, such as gender and socioeconomic status, is of paramount relevance as it allows the calculation of immunisation indicators across subgroups of the population^35^ and highlights the unmet prevention needs of vulnerable groups. For the generalizability of results, although our evaluation covers the population living in a single city only, the findings can, in part, be indicative of differences in vaccination coverage between Italians and migrants in our country, as the study was based on a vast city (2.7 million inhabitants), and a whole population, and access to vaccination was offered to all residents, both Italians and non-Italians, without restrictions in all the Italian regions.

In conclusion, migrants residing in Rome, Italy, showed a lower uptake of COVID-19 vaccination over the first year of the vaccination campaign, independently of socioeconomic factors. Vaccination uptake was lower among migrant women from central-western Asia than migrant males. Health communication strategies oriented to migrants and considering their different languages, cultures, and health literacy, as well as the possible interactions of the provenience country with gender, should be adopted to prevent and reduce inequalities, preferably before emergencies.

## Methods

### Study design and setting

This retrospective longitudinal study was based on residents in Rome on 31 December 2020, followed-up for vaccination against COVID-19 in 2021. The vaccination campaign in Italy started on 27 December 2020.

### Data sources

We used the Lazio Region Longitudinal Study, the cohort of all residents in the Lazio Region, linked to the CORONAVIRUS surveillance system to count vaccinations and infections (date of vaccination, date of infection), the Regional Health Information System, and the Municipal Registry of Rome to count the resident population registered on 31 December 2020 and 2021 (age, sex, citizenship, death date). All data are anonymized, and the record linkage between archives is possible through an anonymous identifier. The Lazio Region Longitudinal Study is part of the National Statistical Programme, which is approved annually by the Italian Data Protection Authority.

### Participants

Participants were all residents of Rome aged 18–64 years (at entry). The population included migrants, refugees, and asylum seekers as long as they resided in Rome. The age constraint was applied to reduce the risk of selection bias related to the possibility that some individuals got vaccinated against COVID-19 in their country of origin instead of Italy. Such risk would be the lowest at working ages. In addition, there is a lower proportion of migrants in nursing homes and residential care facilities for the aged population (with higher opportunities to get vaccinated) than Italians. Follow-up time started on 27 December 2020 (start of vaccination campaign) and stopped when participants experienced the outcome (first dose of vaccine), died, were lost to follow-up, or the follow-up period ended (31 December 21), whichever happened first. To account for the loss to follow-up, we assigned 6 months of follow-up (until 30 June 2021) to residents identified in December 2020 and missing in December 2021 (loss to follow-up). We did not consider vaccinations that occurred before the start of the vaccination campaign (n = 5). In this study, we assumed those vaccinated between 27 December 2020 and 30 December 2020 were still in the region on 31 December 2020.

### Variables

The outcome was the time to the first dose of vaccine against COVID-19. We considered the migratory status assigned through citizenship (non-Italian vs Italian) as the primary exposure. We grouped the origin countries based on migratory pressure: HMPCs vs Italy and geographical areas (central and eastern Europe, northern Africa, sub-Saharan Africa, Latin America, central and western Asia, and eastern Asia) within HMPCs vs Italy. In addition, within each geographical area, we evaluated the association between sex (females vs males) and vaccination. We grouped all the foreign countries except HMPCs into HDCs. Only for completeness, we showed descriptive statistics for this last group, which was not the focus of our analysis.

Covariates included in the analysis for confounding were age (years) and a census-block level deprivation index (DI) based on census data^36,37^. History of SARS-CoV-2 infection was included as a strata variable to account for different timing of vaccination in relation to past infection. The DI is a composite index based on five indicators of socioeconomic disadvantage (low level of education, unemployment, housing tenure, single-parent families, and residential density (occupants per 100 m^2^). A continuous index is divided into quintiles of population level: the higher the quintile, the higher the deprivation. We calculated the quintiles of the continuous index for the 18–64-year-olds eligible for this study.

### Statistical analysis

We performed descriptive statistics. We calculated crude, age-, and age- and DI-adjusted vaccination rates (VR) using the Poisson regression with an offset variable calculated as the natural logarithm of person-time at risk. We used Cox regression models to estimate adjusted hazard ratios (HRs) of vaccination (event: time to vaccination) for migrants from HMPCs compared to Italians. We also applied the Cox model to estimate HRs for females compared to males among migrants from HMPCs, stratifying by geographical area of origin. The models were adjusted for age and DI, and stratified by infection history. We set confidence intervals for the estimated measures at the 95% level. In an additional analysis, we selected the participants excluding those who had an infection before 27 December 2020, and we truncated the follow-up time at the date of infection for those who got infected in 2021. We used the SAS Enterprise Guide software to perform data extraction and statistical analyses.

### Ethics declarations

Patient consent was waived due to the use of administrative data for the Regional Health Service. The Lazio Region Longitudinal Study is part of the National Statistical Programme (PSN 2017–2019, LAZ-00006), and it is approved annually by the Italian Data Protection Authority. The study was conducted in accordance with the Declaration of Helsinki. The dataset was created under the project “COVID19: epidemiological, clinical, genetic, and social determinants of infection and disease progression” approved and financed by the Italian Ministry of Health in response to the COVID-19 pandemic (COVID-2020-12371675). The study was acknowledged by The National Institute for Infectious Diseases Lazzaro Spallanzani Ethics Committee on 29 July 2020. The data were analyzed anonymously through a standardized methodology according to the Italian national privacy law (national legislative decree on privacy policy no. 196/30 June 2003). The Department of Epidemiology of the Lazio Regional Health Service is the regional referral center for epidemiological research, and it has full access to anonymized health information systems. We declare that all methods were carried out in accordance with relevant guidelines and regulations.

## Data Availability

For privacy reasons, restrictions apply to the availability of the data, and individual data are accessible following strict rules on the Lazio Region servers and cannot be exported. Aggregated data are available from the authors upon request (please contact Nera Agabiti, n.agabiti@deplazio.it).
